# Effect of low climate impact vs. high climate impact inhalers for patients with asthma and COPD-a nationwide cohort analysis

**DOI:** 10.1186/s12931-024-02942-8

**Published:** 2024-09-12

**Authors:** Barbara Bonnesen, Josefin Eklöf, Tor Biering-Sørensen, Daniel Modin, Marc Miravitlles, Alexander G. Mathioudakis, Pradeesh Sivapalan, Jens-Ulrik Staehr Jensen

**Affiliations:** 1 Section of Respiratory Medicine, Department of Medicine, Herlev and Gentofte Hospital, University of Copenhagen, Hellerup, Denmark; 2 Department of Cardiology, Herlev and Gentofte Hospital, University of Copenhagen, Hellerup, Denmark; 3 Department of Biomedical Sciences, Faculty of Health and Medical Sciences, University of Copenhagen, Copenhagen, Denmark; 4 Pneumology Department, Hospital Universitari Vall d́Hebrond, Vall d’Hebron Institut de Recerca (VHIR), Vall d’Hebron Barcelona Hospital Campus, CIBER de Enfermedades Respiratorias (CIBERES), Barcelona, Spain; 5 North West Lung Centre, Wythenshawe Hospital, Manchester University NHS Foundation Trust, Manchester Academic Health Science Centre, Manchester, UK; 6 Division of Immunology, Immunity to Infection and Respiratory Medicine, School of Biological Sciences, The University of Manchester, Manchester, UK; 7 Department of Clinical Medicine, Faculty of Health Sciences, University of Copenhagen, Copenhagen, Denmark

**Keywords:** Asthma, COPD, Climate impact, Exacerbation, Mortality, Admission, Pneumonia

## Abstract

**Background:**

Chronic obstructive pulmonary disease (COPD) and asthma can be treated with inhaled corticosteroids (ICS) delivered by low climate impact inhalers (dry powder inhalers) or high climate impact inhalers (pressurized metered-dose inhalers containing potent greenhouse gasses). ICS delivered with greenhouse gasses is prescribed ubiquitously and frequent despite limited evidence of superior effect. Our aim was to examine the beneficial and harmful events of ICS delivered by low and high climate impact inhalers in patients with asthma and COPD.

**Methods:**

Nationwide retrospective cohort study of Danish outpatients with asthma and COPD treated with ICS delivered by low and high climate impact inhalers. Patients were propensity score matched by the following variables; age, gender, tobacco exposure, exacerbations, dyspnoea, body mass index, pulmonary function, ICS dose and entry year. The primary outcome was a composite of hospitalisation with exacerbations and all-cause mortality analysed by Cox proportional hazards regression.

**Results:**

Of the 10,947 patients with asthma and COPD who collected ICS by low or high climate impact inhalers, 2,535 + 2,535 patients were propensity score matched to form the population for the primary analysis. We found no association between high climate impact inhalers and risk of exacerbations requiring hospitalization and all-cause mortality (HR 1.02, CI 0.92–1.12, *p* = 0.77), nor on pneumonia, exacerbations requiring hospitalization, all-cause mortality, or all-cause admissions. Delivery with high climate impact inhalers was associated with a slightly increased risk of exacerbations not requiring hospitalization (HR 1.10, CI 1.01–1.21, *p* = 0.03). Even with low lung function there was no sign of a superior effect of high climate impact inhalers.

**Conclusion:**

Low climate impact inhalers were not inferior to high climate impact inhalers for any risk analysed in patients with asthma and COPD.

## Introduction

Chronic obstructive pulmonary disease (COPD) and asthma can be treated with inhaled corticosteroids (ICS) delivered by low climate impact inhalers (dry powder inhalers) or high climate impact inhalers (pressurized metered-dose inhalers). High climate impact inhalers contain the potent group of greenhouse gasses hydrofluorocarbons causing a climate footprint approximately 20 times larger than with low climate impact inhalers [1–3]. No studies have been conducted in patients with both asthma and COPD comparing delivery of ICS by low and high climate impact inhalers, though ICS is a central part of the treatment of these patients. Patients with both asthma and COPD are in general not very well-characterized, especially in terms of treatment options, though they comprise a large group of patients worldwide.

The deposition pattern of pharmaceuticals delivered by low and high climate impact inhalers have been examined in small studies [4–6], and the clinical effects and risks of ICS delivered by low and high climate impact inhalers for patients with asthma without COPD and COPD without asthma has been analysed in randomized clinical trials (RCT)s [7–16] and epidemiological studies [17–22]. The results are inconsistent [7–22] with a possible increased effect [17–21] and risk [22] of ICS delivered by high climate impact inhalers. As no studies have depicted the effect and risk profile of ICS delivered by low and high climate impact inhalers for patients with both asthma and COPD, the aim of this study was to examine the beneficial and harmful effects of ICS delivered by low vs. high climate impact inhalers in patients with both asthma and COPD.

Our primary hypothesis was that treatment with ICS delivered by high climate impact inhalers was associated with fewer exacerbations requiring hospitalization and less all-cause mortality than low climate impact inhalers in patients with both asthma and COPD. This would reason the negative climate footprint of inhaler therapy for patients with concomitant asthma and COPD. Our secondary hypothesis was that treatment with ICS delivered by high climate impact inhalers was associated with more hospitalizations with pneumonia.

## Methods

### Study design

A nationwide retrospective cohort study was conducted by combining information from the following registries:

1. The Danish Register of Chronic Obstructive Pulmonary Disease (DrCOPD): A nationwide database established in 2008 containing information on the quality of treatment of all patients with COPD treated by a respiratory medicine specialist at a Danish Hospital in an out-patient clinic [23]. Covariates included in this study were smoking status, dyspnoea assessed using the Medical Research Council (MRC) Dyspnoea Scale, BMI (body mass index) assessed as kilograms per square meter, and lung function assessed as forced expiratory volume in the first second as percent of predicted (FEV_1_%) [24].
2. The Danish Civil Registration System: All citizens in Denmark acquire a unique personal identification number at birth or immigration. This unique personal identification number yields data on date of birth and gender, and links individual information for each resident in all Danish registries [25].
3. The Danish National Health Service Prescription Database holds information on all prescriptions dispensed by Danish pharmacies since 1994 (coded according to ATC classification), including date of dispensation, quantity dispensed, strength, and formulation. All pharmacies are required by Danish legislation to provide information that ensures complete and accurate registration. [26]
4. The Danish National Patient Registry holds information on all admissions to Danish hospitals since 1977, and hospital outpatient clinic visits since 1995. Each visit is coded by physicians with one primary diagnosis and one or more secondary diagnoses, according to the International Classification of Diseases, eighth revision (ICD-8) codes until 1994 and ICD-10 thereafter [27].
5. The Danish Register of Cause of Death holds information on all deaths of citizens, who have died in Denmark since 1875 [28] coded according to ICD-8 classification until 1994 and ICD-10 thereafter.

### Population

All Danish residents with a respiratory medicine specialist verified diagnosis of asthma (DJ45 or DJ46) and COPD (as registered in the DrCOPD database) seen in an outpatient clinic from January 1st, 2010, to March 14th, 2022 were included. Hence, the inclusion criteria were a diagnosis of asthma, a diagnosis of COPD and contact to an outpatient clinic. The diagnoses were registered by respiratory medicine specialists in relation to outpatient clinic contacts. Exclusion criteria were collection of ICS delivered by both high and low climate impact inhalers simultaneously, no collection of ICS, and one patient was excluded as his or her inhaler type could not be specified. We compared ICS delivered by low or high climate impact inhalers in patients with both asthma and COPD.

A patient’s study entry date was defined as 365 days after the date of their first collection of ICs delivered by a low or high climate impact inhaler, appendix I for an overview of ATC codes. All patients who collected ICS delivered by both formulations, were excluded. Only patients collecting ICs delivered by low or high climate impact inhalers were included in the propensity score matched cohort.

The patients who did not collect ICS treatment of any kind were included in the sensitivity analyses. In their case, study entry date could evidently not be their first collection of ICS, and hence their date of entry was defined as the date, when they had been treated and monitored by a respiratory medicine out-patient clinic for the same time frame, as the average patient collecting ICS.

The study period ran from January 1st, 2010, to March 14th, 2022 as we considered treatment and monitoring before 2010 dated and follow-up was limited to the time of conducted analysis (April 2023).

### Baseline characteristics

All baseline characteristics were assessed from The Danish National Patient Registry and The Danish National Health Service Prescription Database.

### Follow-up

Patients were followed for one year from study entry. This time frame served as the study period, during which patients were eligible to develop an event. There were no censoring criteria for the primary analysis, however for secondary analyses all-cause mortality was treated as a competing risk, except for the secondary analysis of all-cause mortality.

### Outcomes

All outcomes were assessed for one year. During follow-up patients were followed for events of the primary outcome; composite outcome of exacerbations requiring hospitalization (admissions diagnosed as DJ44 Chronic obstructive pulmonary disease and all belonging subcodes as both A and B diagnoses) and all-cause mortality, and for secondary outcomes; admission with pneumonia (DJ13-DJ16, DJ170, DJ18, DJ2), all-cause mortality, all-cause admissions and exacerbations requiring prescriptions of prednisolone (ATC-code H02AB06 and H02AB07) but not admission. The outcomes were assessed from The Danish National Patient Registry, The Danish National Health Service Prescription Database and The Danish Register of Cause of Death.

### Statistics

Patients treated with ICs delivered by low or high climate impact inhaler were propensity score matched on known and likely confounders; age (as a continuous variable), gender, tobacco exposure (divided into the categories “never smoking”, “passive smoking”, “previous smoking”, “active smoking” and “unknown tobacco exposure”), exacerbations requiring hospitalization in the year before study entry, MRC (with the options 1, 2, 3, 4 and 5), BMI (as a continuous variable), FEV_1_%, collected ICS dose and entry date. All variables for the propensity score match were assessed as close as possible to the entry date; for tobacco exposure the mean assessment took place 1 year and 4.8 months before study entry (IQR 4 years and 2.5 months – 0 years and 2.9 months); for MRC mean 1 year 5.1 months, CI 4 years 2.9 months – 0 years 3.0 months; FEV_1_% mean 1 year 5.6 months, CI 4 years 2.9 months – 0 years 3.1 months, and BMI mean 1 year 5.1 months, CI 4 years 2.5 months – 0 years 3.0 months. These variables are often, but not always updated at out-patient visits. Propensity score matching was performed using the Greedy Match algorithm from the Mayo Clinic [29].

Cox regression model and cumulative incidence curves with Grays analysis was used to assess the risk of events in the compared groups.

Some patients had more than one event during the follow-up period, and in this case only the first event was counted.

For the primary analysis unadjusted Cox regression model was employed.

For sensitivity analysis, we conducted an adjusted Cox proportional hazard regression model of the primary outcome in the unmatched population of all 10,947 patients with asthma and COPD; the 9,785 patients collecting ICS by low or high climate impact inhalers and 1,162 patients who did not collect ICS in the year before study entry. This analysis was adjusted for the variables included in the propensity score match (age, gender, tobacco exposure, exacerbations requiring hospitalization in the year before study entry, MRC, BMI, FEV_1_%, ICS dose and entry date). An additional analysis examined the most vulnerable group of patients with FEV_1_% < 30%.

Model control investigating the proportional hazards assumption was performed to validate the Cox proportional hazards regression, in all cases yielding p values > 0.05.

All statistical analyses were performed using SAS 9.4, Cary, NC, USA, and Microsoft Excel, Windows 365. A two-sided 95% confidence interval was considered statistically significant. Cumulative Incidence plots were customized by the NewSurv macro [30].

Results were presented as hazard ratios (HR) with 95% confidence intervals (CI).

Risks were visualized by cumulative incidence plots with Grays analyses. HR profiles of variables were visualized by forest plot.

### Ethics

The study was approved by the Approval Committee of the Capital Region of Denmark by the Knowledge Centre for Data reviews (P-2022-952). In Denmark, retrospective use of register data does not require ethical approval or patient consent, hence, no patient consents were obtained. This access to data without patient consent is based on the inability to identify any real-life patient from data from the registries, as all data is completely anonymised. The research was conducted in accordance with the Declaration of Helsinki.

## Results

We identified 106,199 patients with COPD diagnosed in relation to a hospital contact. Of these 57,093 were seen by a respiratory medicine specialist in an outpatient clinic, and of these 11,552 patients suffered from both asthma and COPD. Among the patients with specialist verified diagnoses of asthma and COPD 9,785 were treated with a quantifiable prescription of ICS by a low or high climate impact inhaler during 1st January 2010–14th March 2022. Hence, 49,106 patients were excluded as they were not seen in an outpatient clinic and hence did not have specialist verified diagnoses, and 45,541 were excluded as they suffered from COPD without asthma. Only two patients were excluded as they always received ICS delivered by both low and high climate impact inhalers. Further 1,764 were excluded as they did not receive ICS for a full year before entry. One patient was excluded as his or hers ICS could not be specified.

**Fig. 1:**
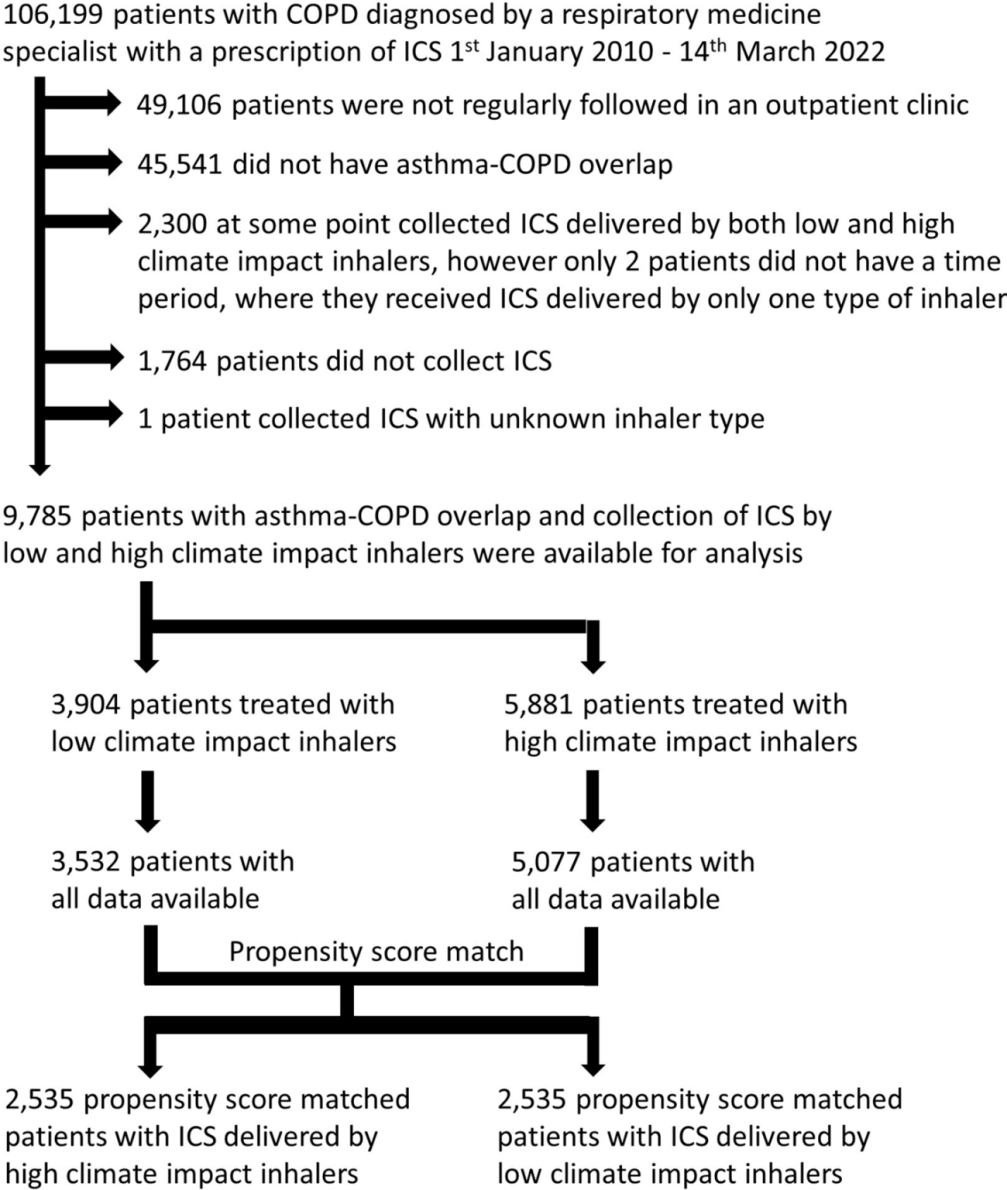
Flowchart of included patients. Included patients were propensity score matched patients 1:1 by age, gender, tobacco exposure, MRC, BMI, FEV_1_%, exacerbations requiring hospitalization and daily dosage of inhaled corticosteroids in the year before entry date as well as entry year. Sensitivity analyses included the 9,785 patients with asthma and COPD who collected identifiable ICS delivered by low and high climate impact inhalers as well as the 926 patients with asthma and COPD, who did not receive ICS in any form

This yielded 9,785 patients available for analysis. They collected 65,697 prescriptions with maximum 69 collected ICS prescriptions for one patient in the 365 days before entry. Of the 9,785 patients, 3,904 collected ICS delivered by high climate impact inhalers and 5,881 patients collected ICs delivered by low climate impact inhalers. For the primary analysis only patients with all data on all variables included in the propensity score match were included (ICS by high climate impact inhalers: *N* = 3,532 and ICS by low climate impact inhalers: *N* = 5,077), Fig. 1.

### Loss to follow-up

Loss to follow-up due to emigration from Denmark was seldom in all investigated groups: In total *N* = 5 patients in the primary analysis were lost to follow-up.

### Baseline characteristics

Propensity score match yielded 2,535 + 2,535 comparable patients treated with ICS delivered by low and high climate impact inhalers, Table 1. Baseline variables were comparable among matching parameters; age 71.5 (ICS delivered by high climate impact inhaler) vs. 71.7 (ICS delivered by low climate impact inhaler), sex (61.5% female vs. 62.6%), tobacco exposure (35.8% active smokers vs. 36.0%), MRC (3 vs. 3), BMI (26 vs. 26), and GOLD FEV_1_% (15.9% stage 4 vs. 15.9%). Similarly comorbidities were very similar between the two groups; Charlson comorbidity index (4 vs. 4), hypertension (29.2% vs. 28.2%), diabetes (13.9% vs. 13.1%) and depression (4.4% vs. 3.9%) as examples. Treatment with other inhaled medicine were also comparable, 97.4% collected prescriptions of long-acting β2-agonist (LABA) among patients treated with ICS by delivered by high climate impact inhalers and 97.5% of patients treated with ICS by delivered by low climate impact inhalers collected LABA for example. Finally, exacerbations requiring admissions and exacerbations treated with prednisolone, but not requiring hospitalization were also similar (25.6% vs. 26.5% and 54.4% and 49.6% respectively).

**Table 1:** Baseline characteristics of the propensity score matched cohorts

Characteristics	Propensity score matched patients with collection of ICS delivered by	
	High climate impact inhalers (pMDI) (*N* = 2,535)	Low climate impact inhalers (DPI) (*N* = 2,535)
Age, years	71.5 (63.5–78.6)	71.7 (64.1–78.5)
Sex, female	1,558 (61.5)	1,588 (62.6)
Entry year	2017 (2016–2019)	2016 (2016–2019)
Tobacco exposure:		
Never smoking	137 (5.4)	170 (6.7)
Previous smoking	1,491 (58.8)	1,452 (57.3)
Active smoking	907 (35.8)	913 (36.0)
MRC	3 (2–4)	3 (2–4)
BMI	26 (22–30)	26 (22–30)
FEV_1_% GOLD stage 4 (FEV_1_% < 30%)	49 (36–63) 403 (15.9)	48 (35–63) 402 (15.9)
Comorbidities within 5 years prior to study entry:		
Charlson comorbidity index	4 (3–5)	4 (3–5)
Hypertension	740 (29.2)	716 (28.2)
Hypercholesterolemia	231 (9.1)	238 (9.4)
Atrial fibrillation	411 (16.2)	390 (15.4)
Diabetes	352 (13.9)	332 (13.1)
Osteoporosis or osteopenia	795 (31.4)	706 (27.9)
Renal insufficiency	130 (5.1)	119 (4.7)
Liver failure	35 (1.4)	47 (1.9)
Malignancy within five years prior to inclusion	318 (12.5)	366 (14.4)
Atopy or allergy	148 (5.8)	140 (5.5)
Depression	111 (4.4)	99 (3.9)
Exacerbations requiring admission within the last year prior to inclusion	650 (25.6)	673 (26.5)
Prescriptions of prednisolone within the last year prior to inclusion	1,380 (54.4)	1,258 (49.6)
Medical treatment for respiratory disease within the last year prior to inclusion:		
Inhaled corticosteroid (ICS) average daily dose, µg	595 (366–921)	579 (316–920)
Use of long-acting β2-agonist (LABA)	2,470 (97.4)	2,471 (97.5)
Use of long-acting muscarinic receptor antagonist (LAMA)	2,185 (86.2)	2,038 (80.4)
Use of short acting β2-agonist (SABA)	2,179 (86.0)	2,069 (81.6)
Use of short acting muscarinic receptor antagonist (SAMA)	393 (15.5)	336 (13.3)

Propensity score matched patients with collection of ICS delivered by high and low climate impact inhalers

Patients were propensity score matched 1:1 by age, gender, tobacco exposure, exacerbations requiring hospitalization, MRC, BMI, FEV_1_% and daily dosage of inhaled corticosteroids in the year before entry date as well as entry year

Characteristics are presented as medians and absolute numbers as relevant with interquartile ranges and percentages in parenthesis

### Primary outcome analysis

**Fig. 2:**
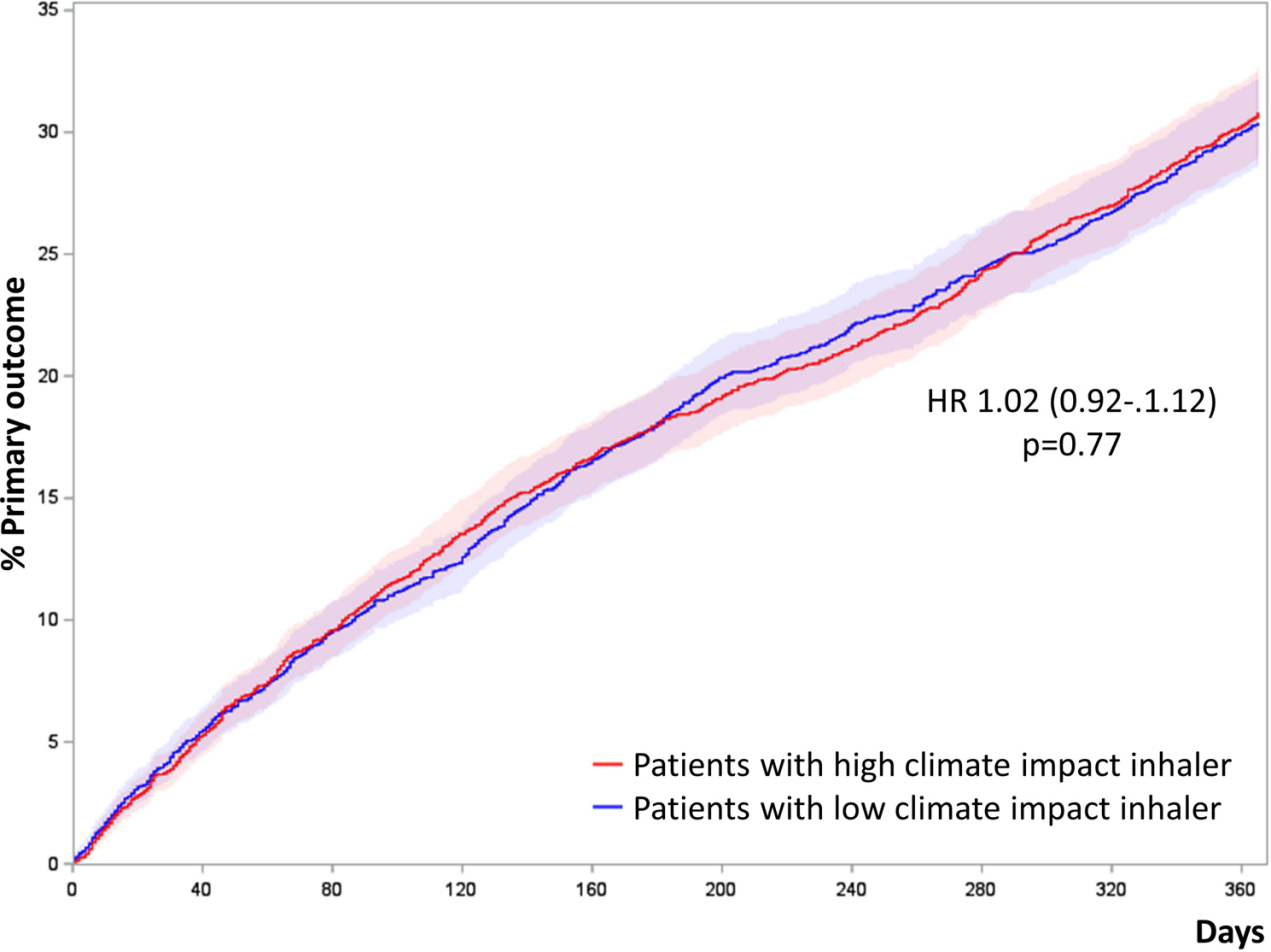
Cumulative incidence plot of exacerbations requiring hospitalization and all-cause mortality in propensity score matched patient groups treated with ICS delivered by low and high climate impact inhalers. Patients were propensity score matched 1:1 by age, gender, tobacco exposure, exacerbations requiring hospitalization, MRC, BMI, FEV_1_% and daily dosage of inhaled corticosteroids in the year before entry date as well as entry year. The primary outcome is a composite outcome of exacerbations requiring hospitalization (admissions diagnosed as DJ44 Chronic obstructive pulmonary disease and all belonging subcodes) and all-cause mortality

Treatment with ICS delivered by high climate impact inhalers was not associated with a lower or higher risk of the primary outcome of exacerbations requiring hospitalization and all-cause mortality compared to ICS delivered by low climate impact inhalers (HR 1.02, CI 0.92–1.12, *p* = 0.77), Table 2 (unadjusted Cox analysis) and Fig. 2 (cumulative incidence plot) among propensity score matched patients.

**Table 2:** Primary outcome

**Outcome**	Propensity score matched patients with collection of ICS delivered by	
	High climate impact inhalers (pMDI) **(*N*** **= 2,535)**	Low climate impact inhalers (DPI) **(*N*** **= 2,535)**
**Primary outcome**		
Exacerbations requiring hospitalisation and all-cause mortality		
*N* (%)	781 (30.8)	770 (30.4)
^#^HR	1.02 (0.92–1.12)	Reference
**Secondary outcomes**		
Admission with pneumonia		
*N* (%)	417 (16.4)	438 (17.3) Reference
^#^HR	0.95 (0.83–1.09)	
All-cause mortality		
*N* (%)	303 (12.0)	287 (11.3)
^#^HR	1.06 (0.90–1.25)	Reference
Exacerbations requiring hospitalisation		
*N* (%)	628 (24.8)	622 (24.5)
^#^HR	1.01 (0.92–1.12)	Reference
All-cause admissions		
*N* (%)	1,303 (51.4)	1,354 (53.4)
^#^HR	0.94 (0.88–1.02)	Reference
Exacerbations requiring prescriptions of prednisolone but not hospitalisation		
*N* (%)	1,019 (40.2)	945 (37.3)
^#^HR	1.10 (1.01–1.21)*	Reference

#Hazard ratios analysed by unadjusted Cox regression analyses of propensity score matched patients with collection of ICS by high and low climate impact inhalers

Patients were propensity score matched 1:1 by age, gender, tobacco exposure, exacerbations requiring hospitalization, MRC, BMI, FEV_1_% and daily dosage of inhaled corticosteroids in the year before entry date as well as entry year

The primary outcome is a composite outcome of exacerbations requiring hospitalization (admissions diagnosed as DJ44 Chronic obstructive pulmonary disease and all belonging subcodes) and all-cause mortality

Secondary outcomes are admission with pneumonia (DJ13-16, DJ170, DJ18 and DJ2) all-cause mortality, exacerbations requiring hospitalization, all-cause admissions and exacerbations requiring prescriptions of prednisolone but not admission (ATC-codes H02AB06 and H02AB07).

Results are presented as absolute numbers and hazard ratios as relevant with percentages and 95% confidence intervals in parenthesis

*indicates statistical significance > 0.95 by regression analysis

### Secondary outcome analysis

**Fig. 3:**
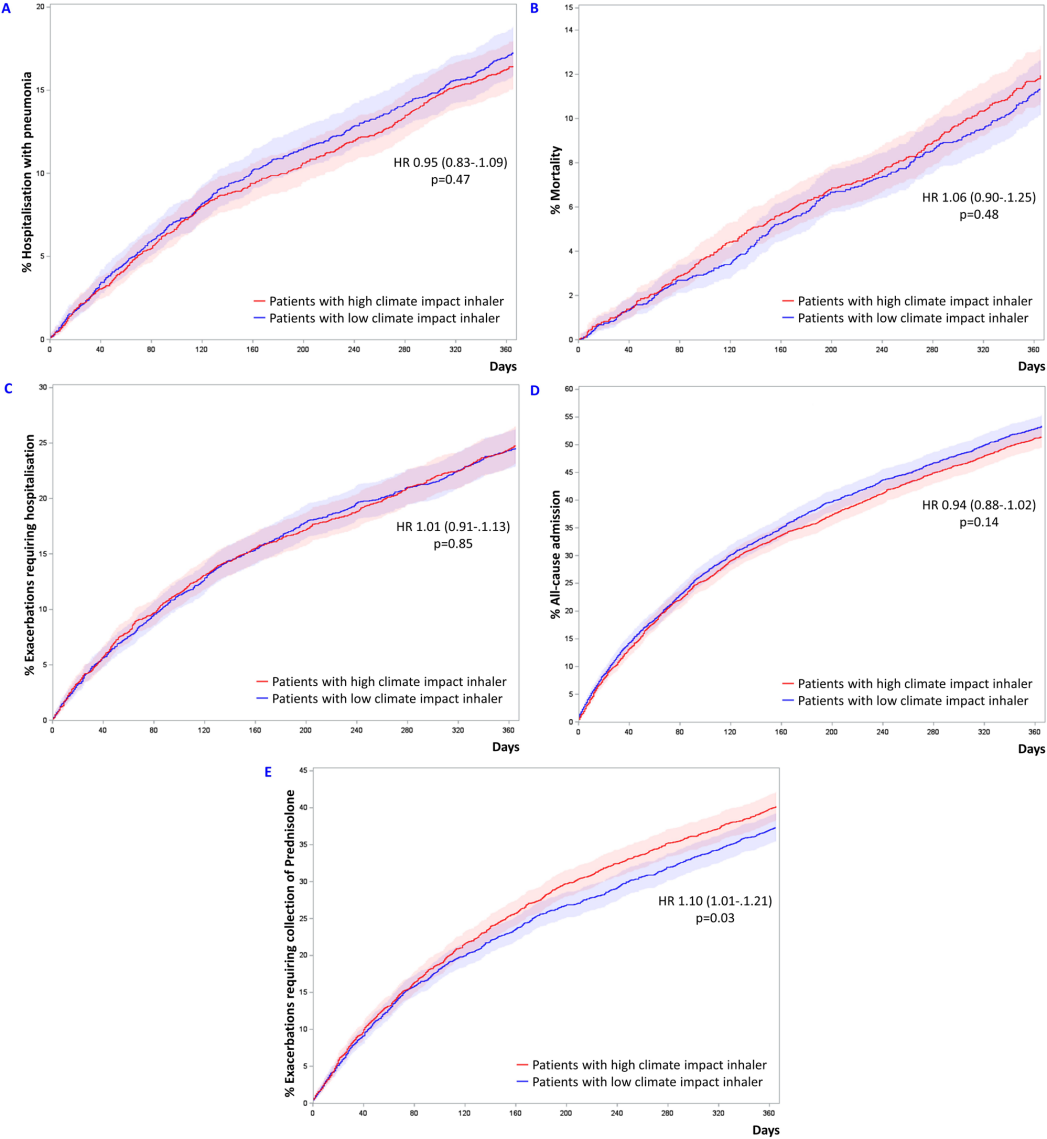
**(A)** Cumulative incidence plot of pneumonia requiring hospitalization in propensity score matched patient groups treated with ICS delivered by low and high climate impact inhalers. **(B)** Cumulative incidence plot of all-cause mortality in propensity score matched patient groups treated with ICS delivered by low and high climate impact inhalers. **(C)** Cumulative incidence plot of exacerbations requiring hospitalization in propensity score matched patient groups treated with ICS delivered by low and high climate impact inhalers. **(D)** Cumulative incidence plot of admission for any cause in propensity score matched patient groups treated with ICS delivered by low and high climate impact inhalers. **(E)** Cumulative incidence plot of exacerbations requiring collection of a prescription for prednisolone but not hospitalization in propensity score matched patient groups treated with ICS delivered by low and high climate impact inhalers. Patients were propensity score matched 1:1 by age, gender, tobacco exposure, exacerbations requiring hospitalization, MRC, BMI, FEV_1_% and daily dosage of inhaled corticosteroids in the year before entry date as well as entry year

Treatment with ICS delivered by high climate impact inhalers was not associated with a higher or lower risk of hospitalization with pneumonia (HR 0.95, CI 0.83–1.09, *p* = 0.47), Table 2; Fig. 3.

Looking at the elements of the primary endpoint, ICS treatment with high climate impact inhalers was also not associated with an altered risk of all-cause mortality (HR 1.06, CI 0.90–1.25, *p* = 0.48), nor was risk of exacerbations requiring hospitalization (HR 1.01, CI 0.91–1.13, *p* = 0.85), or all-cause admissions (HR 0.94, CI 0.88–1.02, *p* = 0.14).

Risk of exacerbation requiring prescriptions but not hospitalization was associated to ICS delivered by high climate impact inhaler vs. ICS delivered by low climate impact inhaler with a HR of 1.10 (CI 1.01–1.21, *p* = 0.03).

#### Sensitivity analysis

Multivariable Cox regression and Forest plot on all patients with asthma and COPD including patients that did not receive ICS therapy.

**Fig. 4:**
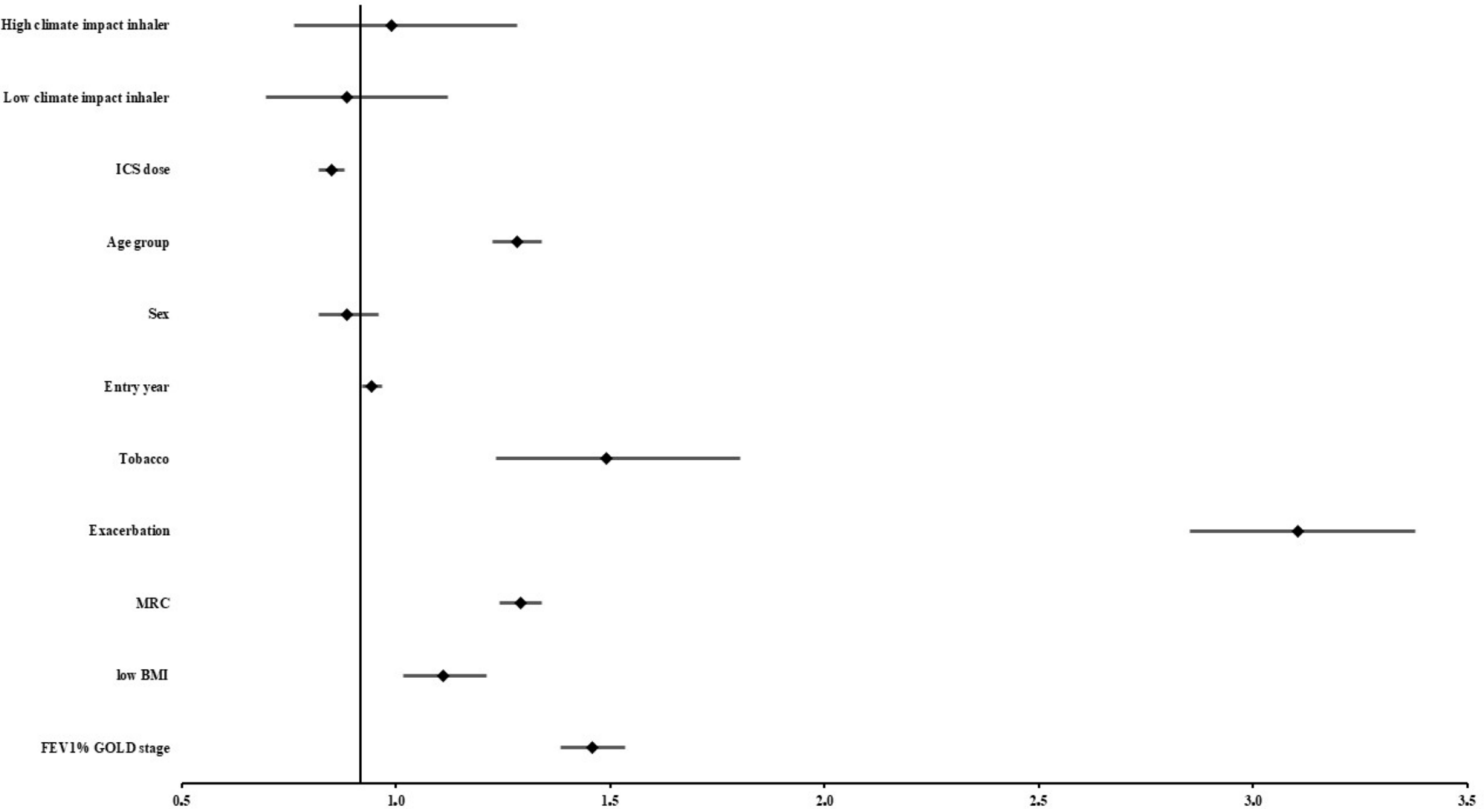
Forest plot of Hazard ratios associated with collection of ICS delivered by low and high climate impact inhalers as well as the variables included in the propensity score match in the unmatched population receiving identifiable ICS delivered by low climate impact inhalers, high climate impact inhalers or no ICS at all. *N* = 10,947 patients with asthma and COPD; 9,785 patients collecting ICS by low and high climate impact inhalers and 1,162 patients who did not collect ICS in the year before study entry. Age group (≤ 70 years, > 70 and ≤ 80 years, > 80 and ≤ 90 or > 90 years), gender (male vs. female), entry year by calendar year, tobacco exposure (active smoking history vs. never smoker), exacerbations requiring hospitalization in the year before study entry, medical Research Council (MRC) Dyspnoea Scale (1, 2, 3, 4 or 5), decreasing body mass index (BMI) (≥ 20 kg/m^2^, < 20 kg/m^2^ and ≥ 15 kg/m^2^ or < 15 kg/m^2^), FEV_1_% GOLD stage 1–4 (≥ 80%, < 80% and ≥ 50%, < 50% and ≥ 30% or < 30%)

A multivariable model analysing the primary outcome (exacerbations requiring hospitalisation and all-cause mortality) and adjusted for inhaler type, and the variables used in the match was performed on the 10,947 patients with all data available. These patients received ICS by high or low climate impact inhaler (9,785 patients), or did not receive ICS (1,162 patients). This yielded a HR of 0.99 for treatment with ICS delivered by high climate impact inhalers (CI 0.76–1.28, *p* = 0.93) and a HR of 0.89 for treatment with ICS delivered by low climate impact inhaler (CI 0.70–1.12, *p* = 0.31), Table 3; Fig. 4.

**Table 3:** Multivariable Cox analysis of the primary outcome on the patients with asthma and COPD who collected identifiable ICS delivered by low climate impact inhalers, high climate impact inhalers or no ICS at all

Variable	Risk of primary outcome: Exacerbations requiring hospitalization and all-cause mortality HR (CI) *p*-value
ICS delivered by high climate impact inhalers	0.99 (0.76–1.28) *p* = 0.93
ICS delivered by low climate impact inhalers	0.89 (0.70–1.12) *p* = 0.31
ICS daily dose in the year prior to study entry	0.85 (0.82–0.88) *p* < 0.0001
Age group	1.28 (1.23–1.34) *p* < 0.0001
Gender male vs. female	0.89 (0.82–0.96) *p* = 0.003
Entry year	0.95 (0.92–0.97) *p* < 0.0001
Tobacco exposure “smoking history” vs. “never smoking”	1.49 (1.23–1.80) *p* < 0.0001
Exacerbations requiring hospitalization in the year before study entry	3.11 (2.85–3.38) *p* < 0.0001
Medical Research Council (MRC) Dyspnoea Scale	1.29 (1.24–1.34) *p* < 0.0001
Decreasing body mass index (BMI)	1.11 (1.02–1.21) *p* = 0.02
FEV_1_% GOLD stage	1.46 (1.39–1.54) *p* < 0.0001

Hazard ratios were calculated by adjusted Cox analysis adjusting for collection of ICS delivered by low and high climate impact inhalers as well as the variables included in the propensity score match. *N* = 10,947 patients with asthma and COPD; 9,785 patients collecting ICS by low and high climate impact inhalers, and 1,162 patients who did not collect ICS in the year before study entry

Age group (≤ 70 years, > 70 and ≤ 80 years, > 80 and ≤ 90 or > 90 years), gender (male vs. female), entry year by calendar year, tobacco exposure (active smoking history vs. never smoker), exacerbations requiring hospitalization in the year before study entry, medical Research Council (MRC) Dyspnoea Scale (1, 2, 3, 4 or 5), decreasing body mass index (BMI) (≥ 20 kg/m^2^, < 20 kg/m^2^ and ≥ 15 kg/m^2^ or < 15 kg/m^2^), FEV_1_% GOLD stage 1–4(≥ 80%, < 80% and ≥ 50%, < 50% and ≥ 30% or < 30%)

Analysis of the matching variables showed HRs of 1.28 (age group increasing risk for each decade, CI 1.23–1.34, *p* < 0.0001), 0.89 (sex decreased risk for men, CI 0.82–0.96, *p* < 0.0001), 1.49 (smoking history with increased risk, CI 1.23–1.80, *p* < 0.0001, however the group of never-smokers comprised only 651 individuals), 3.11 (exacerbations requiring hospitalisation in the year before entry, CI 2.85–3.38, *p* < 0.0001), and 1.29 (increasing severity of MRC, CI 1.24–1.34, *p* < 0.0001), 1.46 (increasing FEV_1_% GOLD stage, CI 1.39–1.54, *p* < 0.0001), 1.11 (low BMI, CI 1.02–1.21, *p* = 0.02), 0.95 (entry year with a slight drop in risk for each calendar year, CI 0.92–0.97, *p* < 0.0001), and finally, 0.85 (ICS dose before entry, CI 0.82–0.88, *p* < 0.0001).

### Risk among patients with FEV_1_% < 30%

There was no interaction between ICS delivered by inhalers with different climate impact and low FEV_1_% (< 30%) in regard to the primary outcome, p for interaction: 0.68.

## Discussion

ICS delivered by low climate impact inhalers was not associated to a higher risk of exacerbations requiring hospitalisation and all-cause mortality than ICS delivered by high climate impact inhalers. ICS delivered by low climate impact inhalers was also not associated to a higher risk of all-cause mortality, exacerbations requiring hospitalisation, or all-cause admission. ICS delivered by low climate impact inhalers was neither associated to a lower or a higher risk of admission with pneumonia. Finally, ICS delivered by high climate impact inhalers was associated to a slightly higher risk of exacerbations requiring a prescription of prednisolone without hospitalisation.

This is the first study comparing COPD exacerbation and death of ICS delivered by low and high climate impact inhalers in patients with both asthma and COPD. Our study is large, has complete follow-up, and available data for controlling several important confounders such at spirometry measures, tobacco history, MRC and BMI. Furthermore, we found no difference in safety profile depending on ICS delivered by low and high climate impact inhalers in patients with asthma and COPD, despite extensive theories on different anatomical deposition patterns, pharmacokinetic properties and device managing [4–6, 31]. This finding should be interpreted in the light of the extensive damage to the climate from high climate impact inhalers [1–3].

The patients in our primary analysis were very closely matched by nine clinical characteristics (age, gender, tobacco exposure, exacerbations requiring hospitalization, MRC, BMI, FEV_1_%, ICS dose and entry date) in order to minimize bias by indication.

Analyses of secondary outcomes similarly revealed no difference in safety profile for ICS delivered by low vs. high climate impact inhalers, with comparable HRs for pneumonia. The analysis on the effect of ICS delivered by high and low climate impact inhalers was elaborated by secondary outcome analyses on all-cause mortality, exacerbations requiring hospitalization or all-cause admission.

One outcome analysis did show an increased risk in patients treated with ICS delivered by high climate impact inhalers: exacerbations requiring prescriptions of prednisolone, but not hospitalization. However, the clinical relevance remains uncertain with a CI almost crossing one. We analysed hospitalisations with exacerbations and hospitalisations with pneumonia as well as exacerbations treated with prednisolone, but we did not analyse exacerbations of concomitant asthma and COPD treated only with antibiotics, as this is not a standard recommended treatment regimen when a patient also suffers from asthma, however, we may have missed some exacerbations / pneumonias not requiring hospitalisation in this context and a difference between groups cannot be ruled out in our study set-up.

A hypothesis has claimed that inhalation medicine delivered by high climate impact inhalers may be a better choice for patients with a low FEV_1_% [32–37]. Therefore, we conducted a sensitivity analysis in which we tested if there was an effect modulation between low FEV_1_% (GOLD FEV_1_% stage 4 with FEV_1_% < 30%) and effect on ICS delivered by low vs. high climate impact inhalers towards the primary outcome. However, this was not the case.

No studies have been performed on treatment with ICS delivered by different types inhaler in patients with both asthma and COPD, however our results are in line with RCTs performed on patients with asthma without COPD and COPD without asthma, which all showed no difference in disease control and safety profile in ICS delivered by high and low climate impact inhalers [7–16]. In contrast to the RCTs, most epidemiological studies in this field have pointed to an improved disease control [17–21] but also an increased risk of pneumonia [22] in patients treated with ICS delivered by high climate impact inhalers.

RCT studies on patients with asthma have shown equal effect and safety profile of ICS delivered by low and high climate impact inhalers in patients with asthma on bronchial hyperresponsiveness, quality-of-life scores, FeNO, spirometry test results, forced oscillation and time to first exacerbation [11–16], whereas epidemiological studies on patients with asthma showed an association between asthma control, reducing asthma exacerbations and FEV1 and treatment with ICS delivered by high climate impact inhalers [17–19, 38]. In patients with COPD, RCTs have shown similar effect on FEV1 [7], but epidemiological studies pointed to a superior effect of ICS delivered by high climate impact inhalers in controlling exacerbations of COPD [20, 21]. Some of the epidemiological studies comparing ICS delivered by low and high climate impact inhalers found a superior effect of ICS delivered by high climate impact inhalers in patients with either asthma or COPD, however this has not been shown for patients with both diseases. Further, several of these studies did not include clinical variables such as tobacco exposure, MRC, BMI and FEV_1_% in their analysis or match [17, 20, 21], one study switched patients from ICS delivered by low to ICS delivered by high climate impact inhalers at the discretion of the treating physician without matching or logistic regression analysis [18], and in one study the ICS treatment by high climate impact inhalers was compared to ICS treatment by low climate impact inhalers in unmatched groups [19]. In an epidemiological study showing an increased risk of pneumonia in patients with either asthma or COPD, matching was performed on only a limited number of variables, and not on important confounders like tobacco exposure, MRC, BMI and FEV_1_% [22].

Our study contributes to the accumulated evidence as both the first study on patients with concomitant asthma and COPD, but also as it is large and very well-matched in comparison to many of the other epidemiological studies done on ICS delivery by high and low climate impact inhalers.

Our study has strengths and limitations. Our study has a its large sample size, complete follow-up, and extensive data availability. The data available to us were extensive and for many patients complete including annually updated data on smoking status, FEV_1_%, MRC and BMI. Our analysis was based on all patients with COPD in an entire country; a population of > 100,000 patients with COPD, of whom almost 10,000 patients had concomitant asthma combined with relevant and quantifiable pharmacological treatment. In this context loss to follow-up was minimal (five patients in total), and the expected impact hereof on the results minimal.

As we were very keen on minimizing the risk of bias by indication, the propensity score matched groups for the primary analysis included only patients with all data available and the groups were matched very tightly on nine different variables. This was successful in generating very comparable groups even on unmatched variables such as co-morbidities evaluated in a hospital setting, which we also have full data availability on. Controlling for important confounders and successful propensity score matching is important to minimize bias by indication. However, our tightly matched groups might pose a challenge for the options for extrapolation of our study on a wider group of patients with asthma and COPD. Our results also appear robust on various outcomes and in both adjusted and unadjusted analyses.

As ICS use by low and high climate impact inhalers was only available to us as collected prescriptions, treatment adherence and inhalation technique were not captured, however since this is a large population, these factors (i.e. non-adherence and insufficient inhalation technique) are likely to be balanced between groups, and, in any case, our results reflect real-life use of both types of devices. We chose to include ICS dose in the matching variables based on collected ICS the previous year. Hence, patients who died just after they collected a first prescription of ICS, were not included and thus a risk of survivor bias cannot be dismissed.

When analysing ICS, it was not possible to consider the different molecule types, nor if delivery was ICS was delivered alongside other pharmaceuticals (LABA or long-acting muscarinic receptor antagonist (LAMA)), as matching or stratifying for these variables would have led to smaller populations for analysis and posed a risk for a type 2 error and a challenge with generalisability. Similarly, it was not possible to match or stratify for the many different types of low and high climate impact inhalers.

As our study is a retrospective cohort study, it has some inherent limitations regarding causality of the findings. However, our study is the first of its kind on the group of patients with both asthma and COPD, it is large and there was complete follow up. Despite the abundant availability of data, some variables were only available as semi-quantified data, and access to more complete data might have improved our analyses.

Future research could explore the development of environmentally friendly inhaler alternatives and assess their impact on patient outcomes and environmental sustainability. Longitudinal studies could also investigate the long-term effects of different inhaler therapies.

In conclusion, ICS delivered by low and high climate impact inhalers do not have different effect or safety profiles, even among patients with low pulmonary function (GOLD FEV_1_% stage 4 with FEV_1_% < 30%). In this light, there is no sign of a clinical benefit which could justify the climate footprint caused by high climate impact inhalers. Evidence-based decision-making should consider both patient outcomes and environmental considerations in inhaler selection.

## Data Availability

All data are available to anyone with genuiene interest though the national database forskermaskinen.
